# Viscous Core Liposomes Increase siRNA Encapsulation and Provides Gene Inhibition When Slightly Positively Charged

**DOI:** 10.3390/pharmaceutics13040479

**Published:** 2021-04-01

**Authors:** Shayan Ahmed, Hugo Salmon, Nicholas Distasio, Hai Doan Do, Daniel Scherman, Khair Alhareth, Maryam Tabrizian, Nathalie Mignet

**Affiliations:** 1 Unité des Technologies Chimiques et Biologiques Pour la Santé (UTCBS), CNRS, INSERM, Université de Paris, F-75006 Paris, France; shayan.ahmed@etu.parisdescartes.fr (S.A.); hai_doan.do@etu.parisdescartes.fr (H.D.D.); daniel.scherman@parisdescartes.fr (D.S.); khairallah.alhareth@parisdescartes.fr (K.A.); 2 Biomedical Engineering Department and Faculty of Dentistry, McGill University, 3775 University St, Montreal, QC H3A 2B4, Canada; hugo.salmon@parisdescartes.fr (H.S.); nicholas.distasio@mail.mcgill.ca (N.D.); maryam.tabrizian@mcgill.ca (M.T.)

**Keywords:** siRNA, neutral viscous core liposomes, positively charged viscous core liposomes, microfluidic device, 3D cell cultures, encapsulation efficiency, loading content, gene delivery, dimyristoylaminopropylaminopropyl, poly(ethylenimine)

## Abstract

Since its discovery, evidence that siRNA was able to act as an RNA interference effector, led to its acceptation as a novel medicine. The siRNA approach is very effective, due to its catalytic mechanism, but still the limitations of its cellular delivery should be addressed. One promising form of non-viral gene delivery system is liposomes. The variable and versatile nature of the lipids keeps the possibility to upgrade the liposomal structure, which makes them suitable for encapsulation and delivery of drugs. However, to avoid the limitation of fast release for the hydrophilic drug, we previously designed viscous core liposomes. We aimed in this work to evaluate if these viscous core liposomes (NvcLs) could be of interest for siRNA encapsulation. Then, we sought to add a limited amount of positive charges to provide cell interaction and transfection. Cationic lipid dimyristoylaminopropylaminopropyl or the polymer poly(ethylenimine) were incorporated in NvcL to produce positively charged viscous core liposomes (PvcL) by a customized microfluidic device. We found that NvcLs increased the encapsulation efficiency and loading content with regards to the neutral liposome. Both PvcL_PEI_ and PvcL_DMAPAP_ exhibited transfection and GFP knock-down (≈40%) in both 2D and 3D cell cultures. Finally, the addition of slight positive charges did not induce cell toxicity.

## 1. Introduction

Viral vectors constitute the majority of gene delivery vehicles in the clinic [1,2]. Despite the fact that synthetic non-viral vectors are potentially less costly to produce and eventually safer, their transfection ability often remains a limitation to effective gene delivery [3]. The major goal for an increasing number of studies has thus been to render non-viral vectors more efficient and to understand their limitations such as biodistribution, cell internalization, nucleic acid release or nuclear delivery [4,5]. The discovery of siRNA and improvement in mRNA production and in vivo use have re-invigorated the field, as their site of action is in the cytoplasm, which significantly reduces the main limitation of nuclear passage needed for DNA therapeutics. This led to the success of the first liposome-based siRNA therapy used for treating polyneuropathy associated with hereditary transthyretin-mediated amyloidosis (hATTR), which was approved by the FDA in 2018 [6]. Another important limitation addressed by hATTR formulation is the lipid chosen. The majority of non-viral vectors which are already described in the literature are positively charged for gene delivery including, liposomes, polymers, nanocrystals, inorganic materials, micelles, and proteins in order to facilitate their interaction with anionic nucleic acids and to provide cell interaction and internalization, which will lead to better transfection efficiency [7,8,9]. However, this cationic charge can impair their circulation time, intracellular release efficiency, and cause toxicity [10,11,12]. The titratable lipids developed more recently were shown to produce significantly long-circulating lipoplexes with reduced toxicity and nucleic acid delivery at a lower pH [13].

After several attempts to reduce the charges of lipoplexes [14,15,16], we sought to formulate lipidic self-assembled nanoparticles with a viscous internal polymeric core, herein referred to as neutral viscous core liposomes (NvcLs). Thickened core liposomes have been proposed before [17]. These studies showed that these liposomes exhibit improved stability and higher encapsulation rate compatible with drug delivery. Moreover, the intertangled polymers forming the liposome core induced a delayed drug delivery [17]. This approach for producing NvcLs nanoparticles with low polydispersity and their thorough characterization of the poloxamer polymer amount within the liposomal core, is the only one reported so far in the literature [18]. These hybrid nanoparticles were prepared with FDA-approved phospholipids and poloxamer to reduce the risk of toxicity and improve their potential for clinical translation. We have shown that small molecules such as rhodamine and doxorubicin could be encapsulated into this novel type of viscous core liposomes with an efficiency of 75% [18,19,20]. To expand the application potential of these thickened core liposomes to the widely growing field of RNAi therapeutics, in this paper, we evaluated the potential of neutral viscous core liposomes (NvcLs) to encapsulate siRNA. SiRNA was encapsulated through a home-made ethanolic injection microfluidic device adapted from our previous study, which allowed us to obtain hybrid lipid polymer nanoparticles [18]. Then, in order to provide cell interaction and transfection, we sought to insert a minimal amount of cationic charges to avoid toxicity. We have developed two strategies to provide the necessary cationic charge to the formulation. The first strategy consisted of providing low cationic charges to the lipidic part of the liposome by the addition of 2-{3-[bis(3-aminopropyl) amino] propylamino}-N-ditetradecyl-carbamoyl methyl acetamide or dimyristoylaminopropylaminopropyl (DMAPAP) as the cationic lipid [3], and the second was to add a low amount of cationic polymer poly(ethylenimine) to the polymer part of the formulation, entrapped in the core of the liposomes [21,22]. Both lipid (DMAPAP) and polymer (PEI) have been shown to allow the internalization of nucleic acid into the cells, leading to high transfection efficiency [23,24]. Herein, we report the entrapment of siRNA in neutral viscous core liposomes (NvcLs) and the transfection efficiency of slightly positively charged viscous core liposomes (PvcLs) in a 2D cell culture, and a first proof of concept in a 3D custom-made microchip. Finally, we addressed the cytotoxicity of the PvcL formulations on endothelial cells.

## 2. Materials and Methods

### 2.1. Materials

DOPC (1,2 dioleoyl-*sn*-glycero-3-phosphocholine) was from Avanti polar lipids (Alabaster, AL, USA), poloxamer 407 (P407) was provided by BASF (Ludwigshafen, Germany), and branched polyethylenimine, PEI was provided by Sigma-Aldrich (St. Louis, MO, USA). The cationic lipid DMAPAP (2-{3-[Bis-(3-amino-propyl)-amino]-propylamino}-*N*-ditetradecyl carbamoyl methyl-acetamide or dimyristoylaminopropylaminopropyl) was synthesized as described [3]. Chloroform and ethanol were purchased from Carlo-Erba reagents (Val-de-Reuil, France). Ultrafiltration units NANOSEP 300k were from PALL filtron (Rehoboth, MA, USA), polyether ether ketone (PEEK) tubes and T-shaped connectors and ferrules were from IDEX Health and Science (Oak Harbor, WA, USA), microfluidic chips were made out of Flexdym 150 × 150 mm^2^ sheets (Eden Microfluidics, Paris, France). Endothelial cell growth medium 2 and trypsin/EDTA were obtained from Cedarlane Labs (Burlington, ON, Canada). The medium DMEM/F12 was from Thermo Fisher Scientific (Waltham, MA, USA), recombinant murine TNF-alpha from PeproTech (Neuilly-Sur-Seine, France), fetal bovine serum albumin, Embryomax^®^ 0.1% Gelatin Solution, and Penicillin-Streptomycin from Sigma-Aldrich (St. Louis, MO, USA). The EA.hy926 (ATCC^®^ CRL2922™) cell line was purchased from the American Type Culture Collection (ATCC, Manassas, VA, USA).

### 2.2. Preparation of Conventional Neutral Liposomes, Neutral Viscous Core Liposomes, Positively Charged LIPOSOMES, and Positively Charged Viscous Core Nanocarriers with PEI or DMAPAP and siRNA

The conventional neutral liposomes (NL) and neutral viscous core liposomes (NvcL) were prepared by the same procedure as described previously [18]. The positively charged liposomes (PL_PEI/DMAPAP_) and cationic hybrid liposome/poloxamer (PvcL_PEI/DMAPAP_) with PEI and DMAPAP were also prepared with the same method as NL and NvcL, only with the addition of positively charged moieties in the composition. The PLs were prepared by the ethanolic injection method using a syringe pump apparatus (Harvard apparatus PHD 2000). One syringe (1 mL) was filled with an ethanolic solution of DOPC (20 mg/mL, 25.4 µmol/mL) or with DMAPAP and DOPC with a ratio 1:9 (2 mg/mL DMAPAP and 18 mg/mL DOPC), and another one (10 mL) with Milli-Q^®^ water containing siRNA (30 nM) and PEI (1.3% or 30 µg/mL) or only Milli-Q^®^ water containing siRNA (30 nM). Both syringes were fixed on the syringe pump apparatus and the injection speed was set at 0.5 mL/min for the lipid solution and 5 mL/min for the aqueous solution. The apparatus ran for 1 min giving a final volume of 5.5 mL, followed by ethanol evaporation that yielded a final concentration of DOPC (1.8 mg/mL) and DMAPAP (0.2 mg/mL or 10% of the total lipid content) after the ethanol evaporation. PvcLs were prepared using the same procedure as PLs but the aqueous phase was replaced by the poloxamer 407 (P407) solution (5% *w/v* or 50 mg/mL) containing siRNA (30 nM) and PEI (0.06% or 30 µg/mL) (ratio PEI/poloxamer) or only siRNA (30 nM), which flowed into the system with a rate of 5 mL/min. The graphical representation and composition of each formulation is available in Table 1.

### 2.3. Transmission Electron Microscopy

The NL and NvcL formulations were observed by transmission electron microscopy (TEM). Briefly, 10 µL of the sample was deposited on a carbon-coated copper grid, the excess solution was removed after 2 min by a filter paper. This was followed by the application of 5 μL of uranyl acetate 1% to the same grid for 2 min, the excess stain was then removed with a filter paper. The sample is subsequently air-dried at room temperature. The grid placed on a slide was inserted in the microscope. The sample analysis was performed with a JEOL JEM 100 S (JEOL Ltd., Tokyo, Japan) TEM operating at 80 kV. TEM images were captured using an Orius Sc 200 digital camera (Gatan-Roper Scientific, Evry, France).

### 2.4. Dynamic Light Scattering (DLS)

A Malvern Zetasizer Nanoseries Nano ZS (Malvern Instruments, France) was used to determine the mean particle hydrodynamic diameter (Z average), polydispersity index (PDI), and zeta potential. The neutral and cationic formulation diameter and PDI were determined at 25 °C by quasi-elastic light scattering. The scattering was measured at a fixed angle of 173° and positioned at 4.65. As for the zeta potential measurements, a dilution of 1/50 of each formulation was carried out in 150 mM NaCl prior to themeasurements.

### 2.5. Nanoparticle Tracking Analysis

Nanoparticle tracking analysis (NTA) was conducted with a 640 nm laser at *t* = 25 °C (NanoSight 300, Amesbury, UK) to measure the size of PvcLs nanoparticles. Each formulation was diluted by 1/500 in Milli-Q and the measurements were performed in a dynamic flow mode controlled by a built-in syringe pump at level 60. The particle size is reported as the hydrodynamic diameter, determined by following the 2D trajectory of each particle over a tracking time with the NanoSight NTA 3.4 software (NanoSight 300, Amesbury, UK).

### 2.6. Ultrafiltration by Centrifugation

Ultrafiltration columns (Pall Filtron Europe, Postmouth, UK) with 300 kDa molecular weight cut-offs were used to separate free poloxamer and non-encapsulated siRNA in the filtrate from the nanoparticles. The filtration was carried out by centrifugation at 13,000 rpm for 30 min at 4 °C in a refrigerated bench-top centrifuge (Hettich Rotanta 460 RF, Tuttlingen, Germany).

### 2.7. Ribo Green Assay

The 100 µg/mL of siRNA suspension was diluted 50-folds with a Tris-EDTA (TE) buffer to reach a concentration of 2 µg/mL. The series of dilutions (1, 0.5, 0.1, and 0.02 µg/mL) was prepared and 100 μL of each concentration was transferred to a 96-well plate. A total of 100 μL of diluted RiboGreen dye (1:200 dilutions in TE buffer) was added to each siRNA concentration. Fluorescence measurements for the calibration curve were made after 5 min of incubation at ambient temperature using a TECAN instrument at emission wavelength of 525 nm. The quantification of encapsulated siRNA was then carried out using the same procedure discussed above for the calibration curve. After filtration, the siRNA in the filtrate of each sample was quantified and expressed as % using equations.

The following are the loading content and encapsulation efficiency equations:

Loading content (LC%) = ((total siRNA − free siRNA)/(amount of lipid component)) × 100;(1)

Encapsulation efficiency (EE%) = ((total siRNA − free siRNA)/(total siRNA)) × 100.(2)

### 2.8. Cytotoxicity Assay

The immortalized endothelial EA.hy926 cell line, commonly used in various angiogenesis and cancer studies, was chosen for in vitro cytotoxicity assays as they can be transfected more easily than primary endothelial cells [25,26].

Briefly, the cells were grown in DMEM containing 2 mM L-glutamine, 100 U/mL penicillin and 100 μg/mL streptomycin, 10% bovine serum at 37 °C, and under 5% CO_2_. Cells were plated onto 96-well plates at 20,000 cells per well, containing 100 μL of culture medium. Twenty-four hours after plating, 100 μL of medium containing the nanoparticles of interest (final concentrations ranging from 0.06 to 2 mg/mL of DOPC, 1.5–50 mg/mL of P407, 0.9375 to 30 µg/mL of PEI, and 0.00625 to 0.2 mg/mL of DMAPAP) was added to the each predefined well. After 24 h of exposure, the cell viability was evaluated using the MTT test in a microplate reader and recorded the absorbance at 560 nm (Infinite F200 Pro-TECAN). All measurements for different formulations were performed in triplicate for three independent experiments.

The cell viability equation:

Viability % = (Asample − mean (Ablank))/(mean (Asolvent) − mean (Ablank)) × 100%(3)

### 2.9. D Cell Culture, Fixation, Imaging, and GFP Quantification

Each well of a 24-well microplate was seeded at 64,000 cells/mL with mouse primary endothelial cells suspended in the endothelial growth medium (EGM-2). After 2–3 days in culture and at the confluency, the cells were transfected with various formulations of siRNA for 4 h. The cells were then rinsed twice with PBS, were fixed using paraformaldehyde 4%, and were stained with DAPI for nucleus and phalloidin-i594 for actin.

Triple fluorescence measurements were performed on an Eclipse TE2000. Acquisition was performed using the open-source Micromanager 1.4, while the image analysis and quantification of GFP level were achieved using the open-source Fiji ImagJ-win64 and a custom-made script. All the conditions were performed in triplicate for each two sets of experiments. The GFP expression of the cells was quantified by taking the same images as analyzed for the qualitative analysis. The GFP expression was calculated with the help of the Fiji imageJ-win64 software for the confocal images by taking into account the number of cells, while taking each nucleus (DAPI) into consideration. The images were first segmented to isolate the structure of interest and the images were then transformed into binary ones. Finally, the nuclei of the stained cells were selected and the various parameters, such as counts and areas, were determined. These results ensure that the qualitative and quantitative analysis have shown a similar kind of result.

### 2.10. Endothelium-on-Chip 3D Cell Culture, Fixation, Imaging, and GFP Quantification

For the endothelium-on-chip device fabrication, the soft thermoplastic foils were hot-embossed at 170 °C on a metallic mold, which was obtained by welding gauge 25 needles. After demolding, the chip is closed on a thin thermoplastic layer and interfaced using thermoplastic studs. A permanent bonding is achieved by baking the chips at 80 °C for 30 min [27]. The chips are subsequently sterilized flowing 70% *v/v* ethyl alcohol diluted in distilled water, rinsed with Milli-Q water, and then coated with 0.1% *w/v* gelatin as an extracellular matrix in an incubator at 37 °C for 30 min, and were seeded with cells as described in Section 2.9. The chips were flipped several times to ensure a full coverage of the V-shape tubular structure of the device with a monolayer of endothelial cells. The confluent cell treatment with RNA formulations and cell fixation procedure for the fluorescence microscopy analyses were performed as described for the 2D cell culture in Section 2.9.

## 3. Results and Discussion

**Figure 1:**
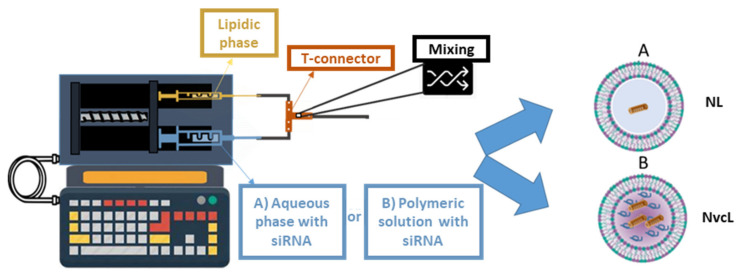
Graphical representation of push syringe technique for the preparation of (**A**). Neutral Liposomes (NLs) and (**B**). Neutral viscous core Liposomes (NvcLs).

Despite the possibility to encapsulate hydrophilic drugs into a liposome core, the applications are rather limited since the equilibrium between the internal and external aqueous media leads to a fast exchange of the hydrophilic drug. This is why, in the last two years, liposomes with increased viscosity into the aqueous core have been designed to enhance the retention of hydrophilic drugs. This current study is the continuation of this recent research work in terms of formulating neutral liposomes (NLs) and hybrid formulation named neutral viscous core liposomes (NvcLs) as a carrier. The idea of replacing the aqueous core with the viscous core was to improve the encapsulation efficiency of the drug in the carrier. We chose to incorporate poloxamer, which is a FDA approved triblock copolymer, into the core. Previous investigations on viscous core liposomes showed a feasible controlled release, as drug encapsulation is enhanced thanks to the viscosity of the core and the drug release requires first the dissolution of the polymeric network [17,18,28]. Moreover, we proposed an automated mixing device to form homogeneous liposomes with the viscous core. As with conventional methods, highly polydispersed liposomes would have formed. This customized microfluidic device was used to mix the microfluidic technology and the ethanolic injection process and produce the formulations shown in Figure 1. Two syringes, containing an ethanolic solution of lipids, and an aqueous or polymeric solution with or without siRNA, respectively were fixed on the microfluidic device and pushed together by the help of a push syringe, as described in Section 2.2 (Figure 1). All the parameters regarding the length of tubing system, diameter of tubing system, injection speed, ratio of lipid and polymer, and their concentrations were studied in detail in our previous publication [18].

In this study, we aimed to adapt the process to encapsulate the siRNA and to form hybrid liposomes encapsulating siRNA in order to answer the following questions: (1) Is it possible to entrap siRNA in neutral viscous core liposomes? (2) Is the device adapted to form positively charged viscous core liposomes? (3) Do slightly positively charged viscous core liposomes allow transfection without inducing cellular toxicity?

### 3.1. Size and Charge Characterization of Neutral Formulations and Their siRNA Encapsulation Efficiency and Loading Content

**Figure 2:**
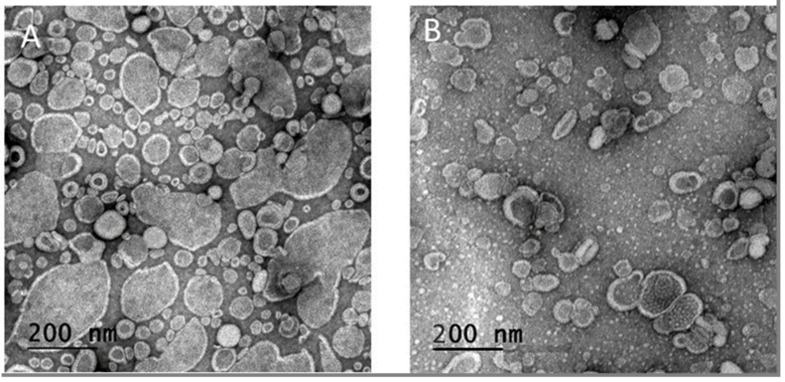
TEM images of neutral formulations. (**A**) NL and (**B**) NvcL formulations.

The first question addressed in this paper was whether increasing the viscosity of the liposome core could entrap siRNA in neutral liposomes. Using the device displayed in Figure 1 and described in Material and Methods, siRNA was added during the process, within the aqueous phase to force its encapsulation during liposome formation. The TEM images showed similar sizes and morphologies for both nanoparticles (Figure 2). The large non-spherical objects in Figure 2a can be the aggregated lipids which fused with each other by drying the TEM grid. Figure 2b shows the NvcLs which are better in terms of their morphologies and sizes, may be due to the stability provided by their inner core. Further detailed characterization with DLS measurements confirmed a similar size for NL and NvcL as shown in Table 2, and a better PDI for NvcL consistent with TEM observations and our previous results [18]. Overall, DLS analyses showed nanoparticles with a PDI of 0.18–0.23 and an average size ranging from of 100 to 125 nm. The particle size distribution curves by the intensity of NL + siRNA and NvcL + siRNA is presented in Figure S2.

To demonstrate that the thicker core of NvcL could improve the encapsulation of siRNA, the free siRNA was eliminated by ultrafiltration and then quantified using the RiboGreen assay. Equations (1) and (2), given in Material and Methods, was then applied and indicated that an encapsulation efficiency (%) and loading content (%) of NL were 8.45 ± 0.21 and 0.016 ± 0.009 and for NvcL 96.7 ± 0.9 and 0.144 ± 0.003, respectively as shown in Table 2. A higher loading content was quantified with NvcL in comparison with conventional liposomes, which we attributed to the viscosity introduced by poloxamer in the self-assembled nanoparticles. To ensure that the encapsulation yield was not artificially increased by the presence of poloxamer within the filtrate, a calibration curve of siRNA was created in the presence of poloxamer and used for the quantification (Figure S1 in Supplementary Data). Furthermore, we quantified the whole passage of free poloxamer by the Baleux assay to ensure that poloxamer would not clog the filter [18].

This is the first example of viscous core liposomes shown to entrap small nucleic acid fragments. In general, nanogels such as the thiol-conjugated hyaluronic acid and disulfide cross-linked dextrin or chitosan particles have been used to encapsulate siRNA or DNA in nanogels. These nanogels obtained by ionic gelation allow an efficient encapsulation of nucleic acids [10,29].

### 3.2. Size and Charge Characterization of Positively Charged Formulations (PvcL)

**Figure 3:**
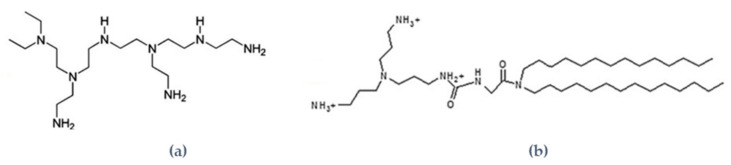
(**a**) Structure of polyethylenimine (branched) and (**b**) structure of dimyristoylaminopropylaminopropyl (DMAPAP) lipid.

**Figure 4:**
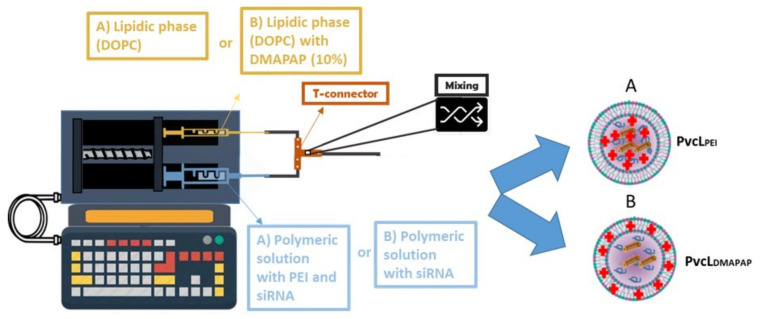
Graphical representation of push syringe technique for preparation of (**A**) PvcL_PEI_ and (**B**) PvcL_DMAPAP_.

Nevertheless, in order to obtain liposomes able to interact with cells, we sought to render the liposomes which are slightly positively charged. As positive charges are used to interact with all surfaces, we wondered if the microfluidic device made with push syringe and T-shaped tubing, made with poly-ether-ether-ketone (PEEK) which is highly resistive and inert, could appropriately allow forming PvcLs. In order to carry out this study the positive charges were added to NL and NvcL formulations via two approaches, either by the addition of PEI (Figure 3a) within the poloxamer polymer phase or by the addition of the cationic DMAPAP lipid (Figure 3b) to the lipidic membrane. The cationic DMAPAP lipid carries ionizable amines on a polyamine moiety which had initially been developed in our laboratory [23]. Both PEI and the DMAPAP lipid have been extensively studied and have been reported to improve the encapsulation efficiency and internalization of nucleic acid into cells [3,21,30,31,32,33]. Using the ethanolic injection and microfluidic device for mixing, the lipids were dissolved in an ethanol solution and the polymers in the aqueous one as shown in Figure 4, according to the amount indicated in Table 1.

In the case of PvcL_DMAPAP_, we added 10% *w/w* of DMAPAP lipid to the neutral lipid, representing only 0.4% *w/w* with regards to the total lipid + polymer weight, in the formulation which is quite lower than what is usually described in the literature where cationic liposomes contain a higher percentage of cationic lipids (approx.: 30–50%) [34,35,36,37].

We used two techniques for size measurement analyses, DLS which measures variations in scattering intensity from a bulk sample, and NTA which measures particle-by-particle mobility related to Brownian motion [38]. In our case both techniques indicated quite monodispersed nanoparticles with a PDI of 0.16–0.22 and a size ranging from 100 to 180 nm typically used for nano-formulations, thereby confirming the suitability of microfluidic method for producing positively charged liposomes (PLs) and positively charged viscous core liposomes (PvcLs) with positively charged moieties such as DMAPAP or PEI (Table 3). The low PDI of PvcL formulations may be due to the presence of poloxamer, which stabilizes and provides a defined shape and size to the liposomes [18]. DLS was also used to measure the electrophoretic mobility and determine the surface charge. As expected, PvcL_PEI_ and PvcL_DMAPAP_ exhibited positive zeta with a value of +8.9 ± 1.8 mV and +5.9 ± 0.9 mV, respectively, whereas the zeta potential was +5.8 ± 1.7 mV for PL_PEI_ and +6.9 ± 1.8 mV for PL_DMAPAP_ (Table 1). These low positive charges are expected to reduce the toxicity without compromising the transfection efficiency, as in literature the high positively charged nanoparticles were reported to cause toxicity issues [7,12,35].

### 3.3. SiRNA Cell Transfection

#### 3.3.1. Cell Transfection in Microplates

**Figure 5:**
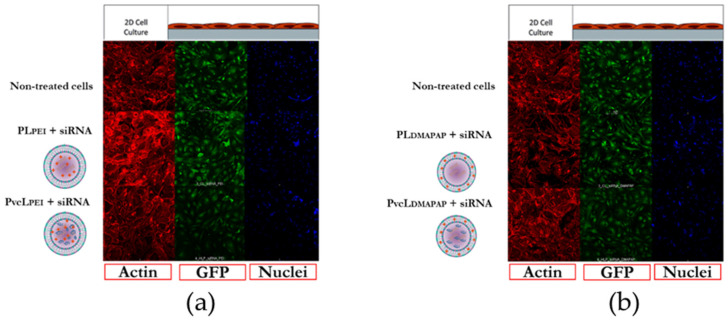
Confocal imaging of endothelial cells treated with various formulations in vitro. (**a**) Non-treated PL_PEI_ and PvcL_PEI_; (**b**) non-treated PL_DMAPAP_ and PvcL_DMAPAP_ treated cells.

The third question we had asked was whether these low positive charged liposomes could be able to deliver their siRNA content into endothelial cells and transfect those cells. The transfection of siRNA was performed in vitro on GFP expressing murine endothelial cells. Confocal microscopy imaging analyses showed the GFP downregulation for cells treated with PL_PEI_ + siRNA, PvcL_PEI_ + siRNA, PL_DMAPAP_ + siRNA, and PvcL_DMAPAP_ + siRNA, when compared with non-treated cells (Figure 5a,b). No changes were observed within the actin filament network, nuclei or cell densities, suggesting that the treatments had no obvious adverse effects on these cells. These results provide a qualitative analysis of gene knockdown efficiency and low cytotoxicity of siRNA encapsulated cationic formulations.

**Figure 6:**
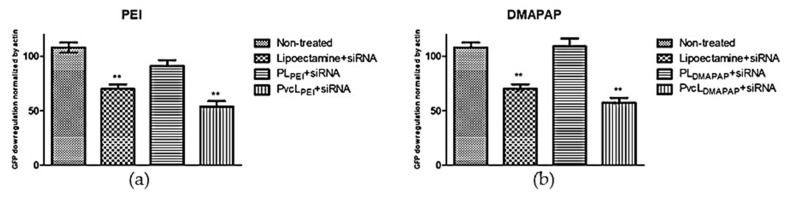
Quantitative analysis of GFP downregulation (normalize by actin) with siRNA encapsulated in cationic formulations. (**a**) Non-treated lipofectamine, PL_PEI_, and PvcL_PEI_, (**b**) non-treated lipofectamine, PL_DMAPAP_, and PvcL_DMAPAP_ treated cells. Student’s *t*-test was applied, *p* < 0.05 (**) for PvcL_PEI_, lipofectamine, and PvcL_DMAPAP_ formulations (*n* = 3).

To further solidify the deduction from the results of confocal images, the quantification analysis was carried out using the software imageJ. The graph (a) in Figure 6 presents the cells treated with positively charged polymer-based formulations; PL_PEI_ + siRNA and PvcL_PEI_ + siRNA in comparison with non-treated cells and PEI + siRNA as a control. The graph (b) in Figure 6 presents the cells treated with positively charged lipid-based formulations; PL_DMAPAP_ + siRNA and PvcL_DMAPAP_ + siRNA in comparison with non-treated and lipofectamine + siRNA taken as a positive control.

Starting from PEI, the liposomes containing PEI taken as a control did show only 90% of GFP inhibition, which was expected for formulations containing only 1.3% PEI. Similarly, the liposomes containing DMAPAP taken as a control showed no inhibition of GFP expression, which was consistent with the fact that only 10% *w/w* of DMAPAP was incorporated into the formulation. Lipidic nanoparticles are less efficient to internalize siRNA into the cells and provide transfection efficiency lower than polymers. This is why other hybrid formulations containing positively charged lipids and anionic polymers were also developed, providing higher stability and transfection ability [39,40,41].

One can see, in Figure 6, that both PvcL_PEI_ + siRNA and PvcL_DMAPAP_ + siRNA were the more efficient formulations tested. They downregulate up to 55% of the GFP expression when compared with non-treated and controls. To note, this inhibition level is obtained for <1% *w/w* positive charge for PvcL_PEI_ and PvcL_DMAPAP_. Indeed, the amount of positive content in the viscous core liposomes is 25 times less in weight due to the amount of incorporated poloxamer within the core. This is important to note as most of the very efficient formulations, described in the literature, present a high positive content, but these cationic liposomes also lead to toxicity issues. Several laboratories have tried to reduce the toxicity by managing the compositions and ratios of the liposomes, some of the examples are DOTAP with carboxymethyl-β-cyclodextrin, DOTAP/Chol/DOPE, and DOTAP/DOPE [35,36].

#### 3.3.2. Cell Transfection Inside the Microchip

In parallel to cell transfection in microplates, we designed a 3D cell culture model in a microchip to test the transfection efficiency of formulations for further applications such as drug development, as 3D models better mimic the natural cell physiological environment [42].

**Figure 7:**
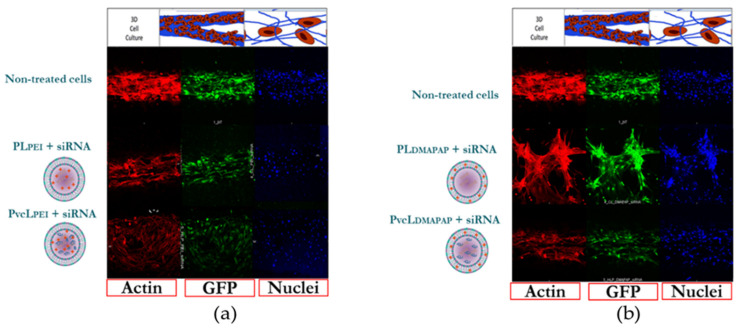
Confocal imaging of endothelial cells in the three-dimensional (3D) culture microchip treated with various formulations in vitro, (**a**) non-treated PL_PEI_ and PvcL_PEI_; (**b**) non-treated PL_DMAPAP_ and PvcL_DMAPAP_ treated cells.

The gene inhibition study was carried out on the same GFP expressing endothelial cells and the same number of cells inside the 3D microchip. Actin and DAPI immunostaining of cells confirmed the formation of a tubular monolayer but their signals varied between different treatments as the 3D nature of the vessels made it more difficult to directly compare fluorescence levels between the samples. This issue made the quantification of GFP expression more difficult. However, the confocal microscopy imaging analyses showed a GFP downregulation for the cells treated with PL_PEI_ + siRNA, PvcL_PEI_ + siRNA, PL_DMAPAP_ + siRNA, and PvcL_DMAPAP_ + siRNA, when compared with non-treated cells (Figure 7a,b). These results should be further confirmed, and the system improved, but these preliminary results are in accordance with the cell transfection obtained in the 2D culture. Interestingly, the 3D cell culture used a very little amount of formulation and medium (~7 µL) representing only 7% of the amount used for cell transfection in 96-well microplates (~100 µL). Therefore, it is highly appropriate for precious samples such as siRNA or mRNA, for instance, and should be further developed.

### 3.4. Cytotoxicity Assay

**Figure 8:**
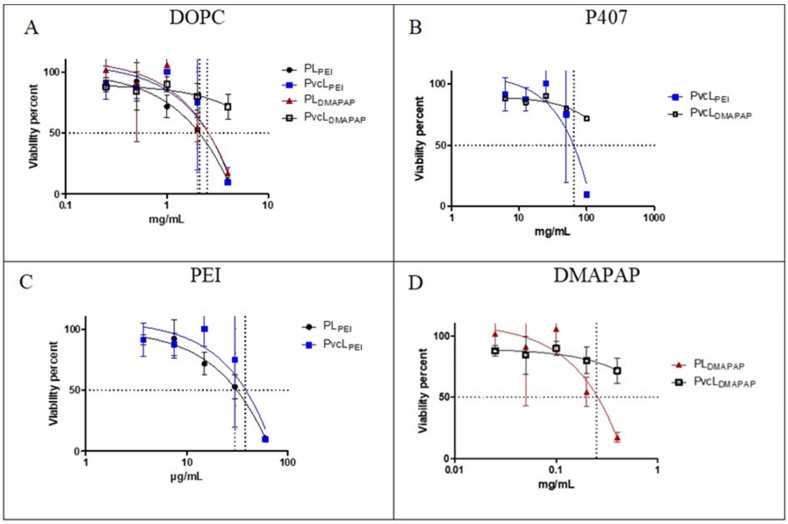
(**A**) DOPC, (**B**) P407, (**C**) PEI, and (**D**) DMAPAP concentration effects on EA.hy926 cell viability. Gaussian nonlinear regression fit and two-way ANOVA was applied *p* < 0.05 (*n* = 3).

Cytotoxicities of the positively charged liposome (PL) and positively charged viscous core liposome (PvcL) formulations with PEI or DMAPAP were evaluated on EA.hy926 cells using the MTT cell viability test. The viability results for PL and PvcL formulations are presented in Figure 8 as a function of lipid (A), P407 (B), PEI (C), and DMAPAP concentrations (D), present in the formulations. The mean inhibition concentration 50 (IC50) values determined for each curve is shown in Table 3. In EA.hy926 cells, the IC50 value was found to be approximately 2 mg/mL for DOPC, 52 mg/mL for P407, 0.03 mg/mL for PEI, and 0.21 mg/mL for DMAPAP, when these moieties were in the PL and PvcL formulations. The PvcL_DMAPAP_ did not show any toxicity at the evaluated concentrations so in this case the IC50 values are not applicable.

The cytotoxicity results obtained for positively charged formulations were similar for PL_PEI_, PvcL_PEI_, and PL_DMAPAP_ when considering DOPC or poloxamer concentrations (Table 4). Noteworthy, the PvcL made with DMAPAP did not show any toxicity in the concentration range studied. The IC50 values were well above the concentrations used in the in vitro experiments. These results indicated no cytotoxic effect within the concentration studied for the transfection evaluation, as the formulations of Table 1 were further diluted into half when added to the culture medium used for cell transfection. These results further highlight their biocompatibility at these concentrations of lipid, poloxamer, PEI, and DMAPAP.

## 4. Conclusions

The present results displayed that the NvcL formulation has better encapsulation efficiency (%) than NL due to the viscous core of liposomes. The addition of slightly positive charges to formulations encapsulating siRNA have efficiently inhibited GFP expression in endothelial cells constitutively expressing GFP. Thus, these PvcLs formulations are able to facilitate siRNA cell internalization without inducing significant toxicity, while releasing the siRNA inside the cells. The DLS and NTA measurements suggest that the optimal size and PDI for the formulations and formulation concentrations are compatible for in vivo use and should be evaluated in the future. The cell culture in the microchip allowed transfecting and imaging cells in a 3D configuration, which is considered a more relevant physiologically environment for investigating various diseases. The 3D cell culture model showed preliminary results consistent with those obtained with the 2D culture, while minimizing the consumption of reagents and medium. Finally, this work highlights the potential of lipid/polymer nanoparticles as a vector for siRNA delivery and potentially other nucleotide-based drugs such as mRNA, for either therapeutic or vaccination purposes. This work suggests the significance of advanced technology such as microfluidics and 3D models for the preparation and the evaluation of innovative therapeutic strategies, including self-assembled nanoparticles.

## Figures and Tables

**Table 1:** Graphical representation of tested formulations and their composition. The siRNA concentration was 30 nM for all the formulations

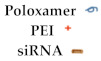	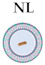	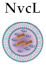	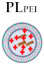	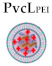	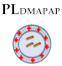	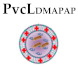
DOPC (mg/mL)	2	2	2	2	1.8	1.8
Poloxamer (mg/mL)	-	50	-	50	-	50
DMAPAP (mg/mL)	-	-	-	-	0.2	0.2
PEI (mg/mL)	-	-	0.03	0.03	-	-

**Table 2:** Characterization of NL and NvcL formulations. Dynamic light scattering (DLS) measurements for size are represented by the mean hydrodynamic average diameter by intensity and the polydispersity index (PDI). This table also presents the encapsulation efficiency % and loading content % of siRNA encapsulated in NL and NvcL. The values and standard deviation are given for nine values, triplicates measured three times each (*n* = 3).

	Size (nm)	PDI	E.E%	L.C%
NL NvcL	104 ± 20 124 ± 15	0.23 ± 0.03 0.18 ± 0.04	8.45 ± 0.21 96.7 ± 0.88	0.02 ± 0.009 0.14 ± 0.003

**Table 3:** DLS and nanoparticle tracking analysis (NTA) analyses for the PL_PEI_, PvcL_PEI_, PL_DMAPAP_, and PvcL_DMAPAP_ formulations. The size is represented by the mean hydrodynamic average diameter by intensity and the polydispersity index (PDI). The values and standard deviation are given for nine values, triplicates measured three times each (*n* = 3).

		DLS	NTA	
	Size (nm)	PDI	Size (nm)	Zeta Potential (mV)
PL_PEI_	97 ± 9	0.34 ± 0.02	180 ± 4.3	+5.8 ± 1.7
PvcL_PEI_	143 ± 12	0.17 ± 0.05	143 ± 1.7	+8.9 ± 1.8
PL_DMAPAP_	113 ± 10	0.25 ± 0.06	172 ± 1.3	+6.9 ± 1.8
PvcL_DMAPAP_	122 ± 16	0.16 ± 0.02	174 ± 2.7	+5.9 ± 0.9

**Table 4:** The mean inhibition concentration 50 (IC50) values for EA.hy926 cells as a function of lipid, P407, PEI, DMAPAP, and siRNA content in the nanocarrier formulations (N/A: Not applicable).

Cell Lines	Component	PL_PEI_	PvcL_PEI_	PL_DMAPAP_	PvcL_DMAPAP_
Endothelial Cells (IC50)	Lipid (mg/mL)	2	˃2	˃2	>5
	P407 (mg/mL)	N/A	52	N/A	>100
	PEI (mg/mL)	0.03	0.03	N/A	N/A
	DMAPAP (mg/mL)	N/A	N/A	0.21	>1

## Data Availability

Not applicable.

## Supplementary Materials

The following are available online at https://www.mdpi.com/article/10.3390/pharmaceutics13040479/s1, Figure S1: Calibration curves for siRNA quantification at different concentrations, (a) siRNA in the absence of P407 and (b) siRNA in the presence of P407. Figure S2: Particle size distribution curves by intensity, (a) NL + siRNA and (b) NvcL + siRNA.
