# Supplementary material for: Viscous Core Liposomes Increase siRNA Encapsulation and Provides Gene Inhibition When Slightly Positively Charged
Source: Pharmaceutics. 2021 Apr 1;13(4):479. doi: 10.3390/pharmaceutics13040479 (PMC8066317; doi:10.3390/pharmaceutics13040479)
Supplement: Supplementary file 1 [file pharmaceutics-13-00479-s001.pdf]

pharmaceutics

MDPI

Supplementary Materials: Viscous Core Liposomes Increase siRNA Encapsulation and Provides Gene Inhibition When Slightly Positively Charged

Shayan Ahmed, Hugo Salmon, Nicholas Distasio, Hai Doan Do, Daniel Scherman, Khair Alhareth, Maryam Tabrizian and Nathalie Mignet *

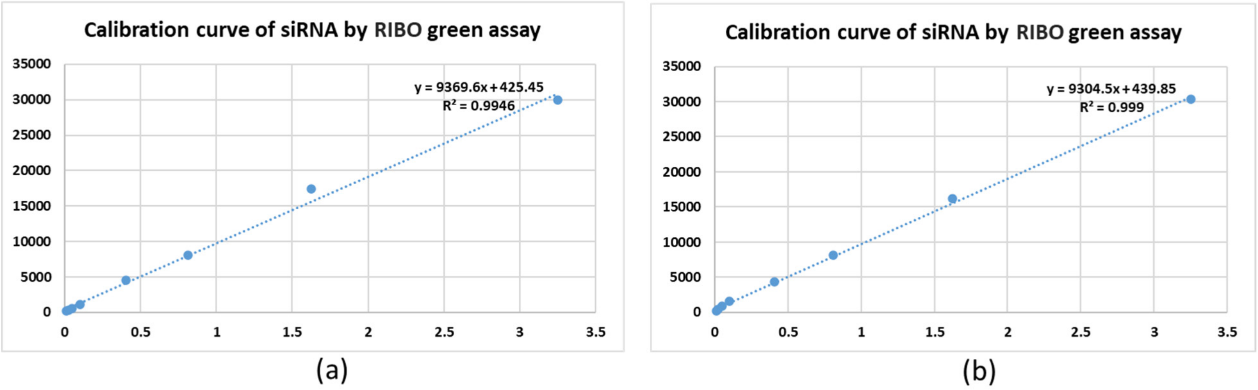

Figure S1. Calibration curves for siRNA quantification at different concentrations, (a) siRNA in the absence of P407 and (b) siRNA in the presence of P407.

Publisher's Note: MDPI stays neutral with regard to jurisdictional claims in published maps and institutional affiliations.

Copyright: © 2021 by the authors. Submitted for possible open access publication under the terms and conditions of the Creative Commons Attribution (CC BY) license (http://creativecommons.org/licenses/by/4.0/).

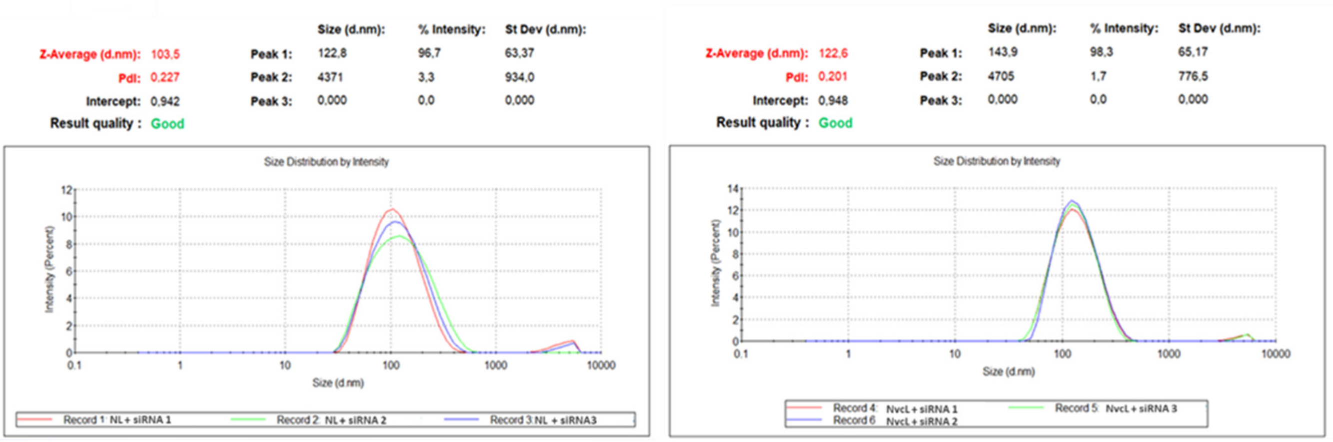

(a) (b)

Figure S2. Particle size distribution curves by intensity, (a) NL + siRNA and (b) NvcL + siRNA.
